# Uses and Perceptions of Music in Times of COVID-19: A Spanish Population Survey

**DOI:** 10.3389/fpsyg.2020.606180

**Published:** 2021-01-12

**Authors:** Alberto Cabedo-Mas, Cristina Arriaga-Sanz, Lidon Moliner-Miravet

**Affiliations:** ^1^Department of Education and Specific Didactics, University Jaume I, Castelló de La Plana, Spain; ^2^Department of Didactics of Musical, Plastic and Corporal Expression, University of the Basque Country, Bilbao, Spain; ^3^Department of Pedagogy, University Jaume I, Castelló de La Plana, Spain

**Keywords:** COVID-19, music, wellbeing, perceptions, Spain

## Abstract

Since March 14, 2020, Spanish citizens have been confined to their homes due to the impact of the COVID-19 pandemic. Participating in musical activities has been associated with reduced anxiety and increased subjective wellbeing. The aim of this study is to analyze how Spanish citizens used music during the lockdown period. We also study perceptions of the impact music has in everyday life, in particular examining the respondents’ insights into the effects of listening to music in situations of isolation. The study was conducted using the MUSIVID19 questionnaire administered to a total of 1868 Spanish citizens. The results indicate that during lockdown, respondents perceived an increase in the time they devoted to musical activities such as listening, singing, dancing or playing an instrument. The participants also reported using music to cope with the lockdown, finding that it helped them to relax, escape, raise their mood or keep them company. The findings suggest an improvement in their perception of the value of music in personal and social wellbeing during the lockdown. However, the study reveals significant differences in the use and perceptions of music according to respondents’ personal situations. Age and feelings of vulnerability may lead to more conservative uses of musical practice and to more moderate perceptions of the positive values of music.

## Introduction

The new COVID-19 pandemic is having a profound effect on the lives of people around the world. Since December 2019, many countries have declared a state of emergency as a result of the health crisis and have imposed various confinement measures on their populations to prevent the spread of the disease. These measures were taken during the middle of March 2020 in most European countries, many of which remain under quarantine at the time of writing. Spain imposed a lockdown on March 16th that confined people to their homes, implemented social distancing measures and closed all businesses, with the exception of those essential to the country’s supply chains (Tobías, 2020). Two weeks later, on March 30th, a more restrictive lockdown was brought in, aimed at reducing mobility and non-essential industrial activity countrywide (Mitjà et al., 2020).

Europe has been heavily impacted by the virus, and this had repercussions not only on citizens’ physical health, but also on social and psychological aspects. Whereas confinement has been shown to have a positive effect in the spread of the disease, it has been reported to have a negative impact on people’s mental health (Liu, 2020; Wang C. et al., 2020; Wang G. et al., 2020; Xiang et al., 2020; Yang et al., 2020). Some of the effects analyzed in people under confinement included confusion, anger, fear, frustration, boredom or post-traumatic stress symptoms (Brooks et al., 2020). Furthermore, confinement causes an increase in negative emotions, such as anxiety, depression and indignation, and of sensitivity to social risks, while decreases are reported in positive emotions such as happiness and life satisfaction (Li S. et al., 2020).

COVID-19 has therefore affected psychological wellbeing. While we acknowledge the complexity of defining the term wellbeing (Ryff, 1989), we understand the construct as a combination of feeling good and functioning effectively in one’s individual and social life (Deci and Ryan, 2008; Huppert, 2009). In Spain, several studies analyzed the impact of COVID-19 on people’s psychological wellbeing during the initial stages of the pandemic. González-Sanguino et al. (2020) and Ozamiz-Etxebarria et al. (2020) conducted online surveys that revealed high levels of depression, anxiety and post-traumatic stress disorders in their samples. Morales-Vives et al. (2020) found variations in the psychological consequences of lockdown according to certain variables, such as gender, age and marital status, and also to personality traits such as subjective happiness, life satisfaction, or higher extraversion. In turn, Rodríguez-Rey et al. (2020) acknowledge the importance of people’s work situation in the psychological impact of the pandemic.

From listening to music to any active participation in music making, individually or in groups, music has been associated with psychological wellbeing, reduced anxiety, lower levels of depression, or coping among people with health conditions and, in general, increased subjective wellbeing (Daykin et al., 2018). Music goes beyond the aesthetic dimension and is part of individuals’ identities and the communicative, cultural, leisure, and psychological aspects of their lives (Fritz and Avsec, 2007; Hormigos-Ruiz, 2010; Cross, 2014; Van der Hoeven, 2018). Participating in any kind of musical experience, in general, and music making, more specifically, can have an impact on positive emotions (Lamont, 2012; Croom, 2015; Campayo–Muñoz and Cabedo–Mas, 2017). These effects can increase when music occurs in a social context (Wuttke-Linnemann et al., 2019). Music has been widely used in psychological and therapeutic interventions to enhance physical and mental health (Peters, 1987; Bunt and Pavlicevic, 2001). The intentional use of music to improve psychological wellbeing has been studied in many areas and with different groups of people, such as those with severe diseases (Chirico et al., 2020; Li Y. et al., 2020), physical impairments (Grau-Sánchez et al., 2020; Hart et al., 2020), various mental health problems (Brancatisano et al., 2020; Greene et al., 2020), relational problems (Dunn et al., 2019; Dvir et al., 2020; Mossler et al., 2020), social or integration difficulties (Crawford, 2017; Henderson et al., 2017; Rodríguez-Sánchez et al., 2018), and also in interventions with different population groups such as pregnant women (Corey et al., 2019; Belloeil et al., 2020), children (Christian Gold et al., 2004; Hallam, 2010) or older people (Coffman, 2002; Creech et al., 2013), among others. Furthermore, for many years (Van de Wall, 1924) music has been used as a tool to increase psychological wellbeing in people experiencing situations of isolation or confinement; this area of interest is currently paramount in psychological and music research (Edri and Bensimon, 2019; Hjørnevik and Waage, 2019; Gold et al., 2020).

Beyond the research experiences of the intentional uses of music for therapeutic purposes, the natural choice to make music in order to raise mood is in many cases an inherent human trait (Chamorro-Premuzic and Furnham, 2007; Storr, 2015). The study of music in everyday life (DeNora, 2000; North et al., 2004) helps us to understand why and how people engage with music, and how participating in musical activities forms part of people’s daily experiences and, therefore, their identities (Frith, 1996). Music may help to consolidate people’s sense of identity, which is built on aspects that include understanding what place music has in people’s lives (DeNora, 2000), how people relate to music (Green, 2011), what the personal and social uses of music are (Small, 1998; MacDonald et al., 2002) and how musical preferences are shaped (Rentfrow et al., 2011). The perceived use people make of music (North et al., 2000; Schäfer et al., 2013) suggests that it is made and consumed naturally to encourage relationships by enhancing the possibility of sharing and connecting with others (Kokotsaki and Hallam, 2007; Cabedo-Mas and Díaz-Gómez, 2013; Schäfer and Eerola, 2020), for cognitive or rational intentions of aesthetic enjoyment (Brattico et al., 2009) and also for emotional regulation (Campayo–Muñoz and Cabedo–Mas, 2017). And in all cases, the musical experience can have an impact on wellbeing.

The few studies that have analyzed the uses of music among the Spanish population have mainly focused on youth and adolescents (Oriola-Requena and Gustems-Carnicer, 2015), or university students (Chamorro-Premuzic et al., 2009). Following initial research by Chamorro-Premuzic and Furnham (2007) on three major uses of music, namely emotional (music for emotional regulation), cognitive (rational appreciation of music) and background (music for social events, work or interpersonal interaction), Chamorro-Premuzic et al. (2009) conducted a study applying the first test of the psychology of music use in Spain to analyze how personal traits influence these uses of music.

The aim of this study is to analyze the uses Spanish people make of music during this period of confinement, and also to explore their views on the impact of listening to and making music on their perceived wellbeing.

## Materials and Methods

### Context and Procedures

The study was largely descriptive in character, since we were interested in collecting data on various aspects, dimensions and components of the studied phenomenon (Hernández et al., 2010). The data were gathered in a Spanish nationwide survey. The research was designed to investigate a series of aspects in relation to the use of music by Spanish people during the period of confinement. The questionnaire – MUSIVID19 – was hosted on a Google Form application, and administered and shared on social media and through university institutional channels. MUSIVID19 was open for responses during the 2-week period between April 3 and 18, 2020.

Researchers from two universities in different regions of Spain designed the questionnaire. Prior to administering the survey, the questionnaire was reviewed by ten experts from the fields of educational research methods, pedagogy, psychology, music and music education. A pilot was conducted with a sample of 20 responses, which led to several changes to the questionnaire before it was finally sent out. MUSIVID19 is provided as Supplementary Material.

**FIGURE 1:**
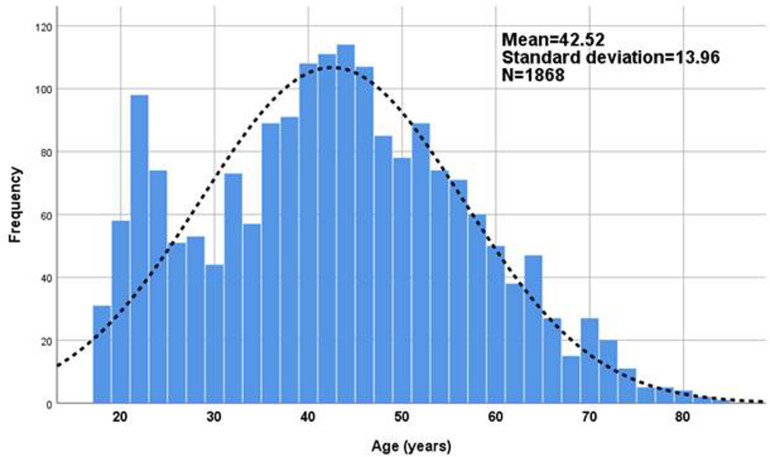
Sample composition according to age (*N* = 1868).

The sample consisted of 1868 participants across all the autonomous communities of Spain. Although we did not control the ways in which the questionnaire was disseminated, the fact that it was accessible across social media, certain web pages and snowballing influenced the randomness of the sample and, therefore, its internal representativeness in relation to different population strata. Although missing data are minimal, around a maximum of 5%, the *N* = 1868 is not constant throughout the study since not all the participants answered all the questions. Of the respondents, 69.3% were women and 30.7%, men. Respondents under the age of 18 were excluded from the sample. Ages ranged from 18 to 84 years old and showed a trend toward the Gaussian distribution (Figure 1). The degree of symmetry is good as demonstrated by the mean age, 42.5 years (CI at 0.95: 41.9–43.2; with standard deviation of ±13.96 years), which is very similar to the median of 42 years. Just over 5% of the participants were over 65 years old; only six people were 80 years old and above.

A large majority of the participants, 88.1%, lived with other people, and a little less than half, 43.1%, with minors. Almost three quarters of the sample, 74.8%, had university education levels, compared to 3.6% of the participants with primary school level or lower. Over half of the participants, 51.9%, were teleworking from home during the period of confinement; a further 10.1% were in face-to-face work, and the rest were not occupationally active for various reasons.

### Measures

In addition to items requesting demographic information, the online survey consisted of three sections: (1) musical identities in confinement; (2) uses and knowledge of COVID-19 musical initiatives and; (3) perceptions about the impact of music. The survey included 22 questions, most of them requiring responses on a 5-point Likert-type scale, and some having a single or multiple choice response format.

#### Musical Identities in Confinement

This section was designed to collect information about the frequency people claim to listen to and participate in music, and about their perceptions of their current use of musical activities in comparison to times of no confinement. The survey gathered information about possible curiosity to broaden musical preferences during confinement by asking whether participants listened to a greater diversity of musical styles or discovered new groups.

#### Uses and Knowledge of COVID-19 Musical Initiatives

This section aimed to collect information about participants’ knowledge and use of the numerous music-related social initiatives that have emerged during the confinement period. The survey asked participants about their level of knowledge of musical projects aimed at promoting personal and social wellbeing led by professional and amateur musicians during this period, such as streaming open concerts, music campaigns, open access platforms, free online music lessons, collaborative music creation initiatives, etc. Information about their uses of these initiatives was also collected to provide a picture of what kind of initiatives were more popular. Lastly, the Spanish people quickly established a ritual applause from their balconies every day at 8 pm to show their gratitude to medical staff. This became an opportunity for people to share musical initiatives with the neighborhood from their balconies. The survey collected information about musical initiatives occurring in their neighborhoods during the applause and the level of engagement participants had in these activities.

#### Perceptions About the Impact of Music

This last section gathered information on the perceptions participants had about the power of music to promote aspects related to dimensions of wellbeing such as the capacity to enhance relief, trust, reduce anxiety or loneliness, to raise mood, to cope with the situation and to feel connected to other people. The survey also collected data about the impact this situation had on participants’ perception of the value of music, its potential to enrich free time, its influence on wellbeing, and the social value of musicians and the role of music in education.

### Data Analysis

Comparative descriptive and inferential statistics were applied to analyze the questionnaire. The chi-square test was used to determine the existence of significant differences between the categorical variables. In addition, the effect size calculation was performed as an indicator of the magnitude of these differences. To do this, the effect size through the *R*^2^ scale was estimated from the square of Cramer’s W Index. In the variables with a Likert response format, the contrast was carried out with the parametric method ANOVA. The effect size value was also calculated, as a means to assess the magnitude of the differences and to allow their comparison with those found in other procedures. This was achieved by expressing this effect size in the form of *R*^2^, as before, which is now obtained from Cohen’s “d” conversion equation.

In the results section we focus only on aspects of the analysis of the data from MUSIVID19 that yielded significant results. The analysis also led to interesting findings when gender, level of education or home situation were controlled for, although these aspects go beyond of the scope of this paper.

## Results

### Descriptive Statistics

In the first section of the questionnaire, musical identities in confinement, and specifically from responses to item 1 (During this period of confinement, how often do you listen to music?), we observed that the vast majority of participants, 83.6% (*N* = 1561), reported listening to music every day during this period. In contrast, only 1.8% (*N* = 34) did so less than once per week, along with another 4.6% (*N* = 85) who said they listened to music 1 or 2 days per week. Among those who responded that they listen to music every day (item 2: If you selected “Every day,” approximately how many hours a day?), 45.6% (*N* = 712) selected between 1 and 2 h a day, followed by 31.3% (*N* = 489) who listen to music between 3 and 5 h a day. The two extreme categories, with the most and the least listening time, accounted for 11 and 12%, respectively. Regarding item 3 (During this period of confinement, have you listened to more diverse styles of music?), 73.6% reported listening to the same music as before confinement; the rest of the sample (26.4%) had discovered new musical styles. In relation to whether they had discovered new musical groups (item 4: Have you discovered new musical groups that you like during this period?), regardless of their previous answer, more than half of the participants (53.1%) affirmed that they had discovered new musical groups that they enjoyed listening to during this period.

In the same section dealing with perceptions about the uses of music, we reviewed the items related to different activities before and after confinement. The results suggest that almost all the respondents listened to as much music or more than before confinement (item 5: Consider that you listen to music…). Specifically, 49.7% say they listened to more music; only 3.7% mentioned that they listened to less. In turn, 8.7% reported singing less than before (item 6: Consider that you sing…). The rest sang as much (the majority, 65.0%) or even more than before confinement (26.3%). Regarding dancing (item 7: Consider that you dance…), 31.4% of participants danced more during confinement than before. While a little more than half (54.2%) danced as much as before, the rest (14.2%) danced less than previously. In reference to playing an instrument (item 8: Consider that you play an instrument…), more than half of the sample, 56.3%, said their activity had not changed since confinement began, while 30.1% of the participants spent more time performing music.

In the second section of the survey the questions relate to uses and knowledge of COVID-19 musical initiatives. Item 9 (Do you know of any initiatives or projects that musicians have undertaken to share or contribute to wellbeing during this period of confinement?) asks about social music-based projects or initiatives that contribute to wellbeing during the confinement period. Responses showed that 31.1% of the sample enthusiastically followed such initiatives, and another 24.2% were aware of them and had also created or participated in some of them. Only 6.6% had no knowledge of these projects. Regarding music performed in their neighborhood (item 10: In your neighborhood, are people making music on their balconies?), more than half of the sample (53.5%) reported that their neighbors were making music on the balconies.

The last section is related to perceptions about the impact of music. Regarding respondents’ perceptions of the value of music during confinement, slightly more than half of the participants (53.1%) stated that music greatly helped them to relax (item 11: Do you think music helps you relax?), and 26.8% considered it helped quite a lot; only 1% said that music did not help them to relax at all. Similar results emerged in responses to the following item, in which 56.0% of the respondents stated that music definitely gave them a way of escaping (item 12: Do you think of music as a form of escape?); and 25.2% answered that it helps them quite a lot. Along the same lines, just 1% responded negatively. Regarding the potential of music to affect mood (item 13: Do you think music cheers you up?), 62.9% reported that music greatly helped to cheer them up, and another 21.9% felt it helped them to positively change their mood. Only 0.2% answered that it did not help them at all. We found similar results in the following question (item 14: Does music keep you company?), to which 58.1% of the sample responded with the maximum score, and another 24.7% answered that music kept them company quite a lot. Regarding capacity to deal with the situation (item 15: Does music help you cope better with confinement?), more than half of the group (51.5%) indicated that music was a great help in coping with confinement, and 27.0% answered that it helped them a lot. Moreover, 77.4% of the sample reported that music boosted their confidence or sense of positivity (item 16: Does listening to music boost your confidence and positivity?), scoring 4 or 5 points in the question. Finally, slightly less than half of the participants (46.9%) stated that sharing music with other people made them feel much more connected (item 17: Does listening to or making music with other people make you feel more connected to them?) with these people, whereas 27.3% said it made them feel quite a lot more connected.

Continuing in the line of enhanced perceptions of music in confinement, (item 18: Do you think confinement has improved your perception of the value of music?), the most frequent responses were values 5 (very much) and 3 (neutral) (30.8 and 28.1%, respectively). For this reason, the average (3.48) suggests that confinement has led to a medium to high improvement in the perception of the value of music. Regarding the value of the work musicians do (item 19: Do you think confinement has improved your perception of the value of musicians’ work?), values 5 (33.8%), 3 and 4 were the most frequent responses; we can therefore conclude that the average (3.59) indicates a medium to high improvement in the perception of the work of musicians. As for the role of music in education (item 20: Do you think confinement has improved your perception of the role of music in education?), the distribution of the responses is similar to the previous ones, with 3 and 5 being the most frequently repeated scores. Therefore, again an average value of 3.64 indicates a medium to high perception. As regards the role of music in enriching free time (item 21: Do you think confinement has improved your perception that music enriches your free time?), a slight increase in value 5 responses (39.0%) raises the average to 3.77; it can therefore be claimed that perception of the possibilities of music to enrich free time has improved notably. Finally, regarding the influence on personal wellbeing (item 22: Do you think confinement has improved your perception of the influence music has on your personal wellbeing?), the mean value (3.78) indicates a notable improvement in this perception.

Table 1 summarizes the main results of the descriptive analysis organized by the three sections of the questionnaire.

**TABLE 1:** Descriptive analysis in percentages (%).

Section 1. Musical identities in lockdown					

*Frequency of listening to music*	>*1*	*1–2*	*3–5*		<*5*
Days per week (*N* = 1867)	1.8	4.6	10.0		83.6
Hours per day (*N* = 1560)	11.0	45.7	31.3		12.0

***Uses of music during and before lockdown***	***Less than before***	***The same as before***			***More than before***

Listening to music (*N* = 1852)	3.7	46.6			49.7
Singing (*N* = 1696)	8.7	65.0			26.3
Dancing (*N* = 1692)	14.2	54.3			31.5
Playing an instrument (*N* = 1513)	13.6	56.3			30.1

***Musical diversity***	***No***		***Yes***		

Discovered new musical styles (*N* = 1856)	73.6		26.4		
Discovered new musical groups (*N* = 1847)	46.9		53.1		

**Section 2. Uses and knowledge of COVID-19 musical initiatives**					

	***No knowledge***	***Has heard about***	***Knows and follows***		***Participates***

Knowledge of musical initiatives (*N* = 1857)	6.6	38.0	31.2		24.2
	***No***		***Yes***		
Neighbors making music on balconies (*N* = 1862)	46.5		53.5		

**Section 3. Perceptions of the impact of music**					

***Perceptions of the value of music***	***Not at all***	***A little***	***Sometimes***	***Often***	***A lot***

Helps you relax (*N* = 1835)	0.6	4.4	15.1	26.8	53.1
Helps you escape (*N* = 1820)	1.0	3.8	14.0	25.2	56.0
Improves mood (*N* = 1832)	0.2	3.2	11.8	21.9	62.9
Keeps you company (*N* = 1827)	0.7	4.5	12.0	24.7	58.1
Helps you cope better with confinement (*N* = 1828)	1.9	6.0	13.7	27.0	51.4
Boosts confidence/positivity (*N* = 1812)	1.4	5.3	15.9	27.5	49.9
Improves connection with others (*N* = 1772)	2.8	6.1	16.9	27.3	46.9

***Has lockdown improved perception of*…*?***	***Not at all***	***A little***	***Sometimes***	***Often***	***A lot***

The value of music (*N* = 1812)	10.4	11.6	28.1	18.9	30.8
The value of the work of musicians (*N* = 1808)	9.0	10.3	27.6	19.3	33.8
The role of music in education (*N* = 1813)	8.7	9.9	26.9	17.8	36.7
Music as an enhancer of free time (*N* = 1816)	6.8	7.5	26.3	20.4	39.0
Influence of music on personal wellbeing (*N* = 1832)	6.9	7.3	26.0	19.9	39.9

### Inferential Statistics

As this research is contextualized in the COVID-19 crisis, which has had a profound impact on the economy as well as on physical and mental health, we carried out an inferential study considering respondents’ current employment situation. This analysis showed that current employment circumstances have considerable implications for the subject studied. Employment situation is defined in five categories: two representing people who were in active employment: telework and face-to-face work; and three representing those who are not currently working: unemployed, furloughed, and retired people. People who were on holiday at the time only represented 2% of the sample and generated very low frequency values; they were therefore removed from the sample. Likewise, responses marked “none of the above” (11.6% of the participants) were eliminated as it was an undefined category. Taking this situation into account, and considering that 4.1% of the sample did not answer this question, the maximum N valid for this inferential analysis block is 1537, 82.3% of the total number of participants.

With the factor thus set out, in contrasting the first and second sections of general variables (Tables 2, 3), the results for almost all of them were statistically significant. Table 2 shows information about the first and second sections, except the comparison of the uses of music now and before confinement, for the sake of clarity. The results indicate statistical significance in all the variables, although with small effect sizes, except for the non-significance of neighbors making music on their balconies. As regards the rest, from the least to the most statistical relevance, the factor of musical diversity indicates that retirees, followed by those still active in face-to-face work, tended to listen to the same music as before. Respondents who most listened to music every day were those in the teleworking group, followed by furloughed workers. The furloughed group was the largest in terms of those who had discovered new musical groups they liked, whereas retired participants tended to listen to music they were familiar with and had not explored new styles of music. Regarding the respondents’ knowledge of music projects designed to help enhance wellbeing, the data indicates that retirees were either unaware of them or had only heard about them, whereas teleworking participants had the most knowledge about these initiatives and had also participated in some of them.

**TABLE 2:** Comparative inferential analysis: Musical identities during confinement and uses and knowledge of COVID-19 musical initiatives.

*VARIABLES/Categories*	*Percentage*					*Chi-square test*		Effect size
		
	Unemployed	Furloughed	Retiree	Teleworking	Face-to-face working	Value	*P* value	
***FREQUENCY OF LISTENING TO MUSIC***	*n* = 174	*n* = 116	*n* = 138	*n* = 928	*n* = 180	22.54 *	0.032	0.007
<*1 time/week*	2.3%	0.0%	3.6%	1.5%	3.3%			
*1–2 times/week*	4.0%	8.6%	6.5%	4.2%	4.4%			
*3–5 times/week*	13.2%	7.8%	**14.5%**	8.3%	10.0%			
*Every day*	80.5%	83.6%	75.4%	**86.0%**	82.2%			
***HOURS LISTENING TO MUSIC***	*n* = 140	*n* = 97	*n* = 104	*n* = 798	*n* = 147	16.12 ^NS^	0.186	0.003
<*1 h/day*	9.3%	8.2%	13.5%	11.9%	10.9%			
*1–2 h/day*	45.7%	45.4%	57.7%	44.7%	51.7%			
*3–5 h/day*	33.6%	37.1%	18.3%	32.8%	25.2%			
>*5 h/day*	11.4%	9.3%	10.6%	10.5%	12.2%			
***MUSICAL DIVERSITY***	*n* = 172	*n* = 115	*n* = 136	*n* = 925	*n* = 178	9.99 *	0.040	0.004
*Listens to the same music*	77.3%	65.2%	**80.1%**	73.9%	78.7%			
*Discovered new musical styles*	22.7%	**34.8%**	19.9%	26.1%	21.3%			
***DISCOVERED NEW MUSICAL GROUPS***	*n* = 172	*n* = 115	*n* = 134	*n* = 921	*n* = 180	19.48**	0.001	0.013
*Yes*	50.0%	**60.0%**	35.1%	53.4%	50.0%			
*No*	50.0%	40.0%	**64.9%**	46.6%	50.0%			
***KNOWLEDGE OF MUSICAL INITIATIVES***	*n* = 172	*n* = 115	*n* = 138	*n* = 926	*n* = 179	63.55**	<0.001	0.014
*No knowledge*	6.4%	5.2%	**16.7%**	4.6%	5.6%			
*Has heard about them*	39.0%	42.6%	**50.7%**	37.0%	37.4%			
*Knows and follows them*	33.1%	32.2%	26.8%	29.4%	33.0%			
*Knows and participates in them*	21.5%	20.0%	5.8%	**28.9%**	24.0%			
***NEIGHBORS MAKING MUSIC ON BALCONIES***	*n* = 173	*n* = 116	*n* = 137	*n* = 927	*n* = 180	3.10 ^NS^	0.541	0.000
*Yes*	51.4%	55.2%	47.4%	53.4%	56.7%			
*No*	48.6%	44.8%	52.6%	46.6%	43.3%			

N. S. = Not significant at 5% (*p* > 0.05) * = Significant at 5% (*p* < 0.05) ** = Highly significant at 1% (*p* < 0.01).

*In bold, categories in which significance is appreciated (residual ≥ 2).*

**TABLE 3:** Comparative inferential analysis: Uses of music during and before lockdown.

*VARIABLES/Categories*	*Percentage*					*Chi-square test*		Effect size
		
	Unemployed	Furloughed	Retiree	Teleworking	Face-to-face working	Value	*P* value	
***LISTENING TO MUSIC***	*n* = 172	*n* = 113	*n* = 136	*n* = 925	*n* = 179	18.62 *	0.017	0.006
*Less than before*	4.1%	**8.0%**	2.2%	4.1%	1.1%			
*The same as before*	**54.7%**	36.3%	48.5%	45.9%	50.8%			
*More than before*	41.3%	**55.8%**	49.3%	49.9%	48.0%			
***SINGING***	*n* = 163	*n* = 111	*n* = 84	*n* = 876	*n* = 166	12.10 ^NS^	0.147	0.004
*Less than before*	10.4%	9.9%	11.9%	8.7%	5.4%			
*The same as before*	66.9%	54.1%	67.9%	65.0%	66.9%			
*More than before*	22.7%	36.0%	20.2%	26.4%	27.7%			
***DANCING***	*n* = 162	*n* = 106	*n* = 87	*n* = 875	*n* = 170	19.95 *	0.011	0.007
*Less than before*	16.0%	**23.6%**	13.8%	12.6%	13.5%			
*The same as before*	54.9%	42.5%	**62.1%**	53.8%	61.8%			
*More than before*	29.0%	**34.0%**	24.1%	**33.6%**	24.7%			
***PLAYING AN INSTRUMENT***	*n* = 143	*n* = 96	*n* = 52	*n* = 815	*n* = 140	16.58 *	0.035	0.007
*Less than before*	**22.4%**	12.5%	19.2%	12.0%	14.3%			
*The same as before*	51.0%	56.3%	**63.5%**	56.7%	59.3%			
*More than before*	26.6%	**31.3%**	17.3%	**31.3%**	26.4%			

N. S. = Not significant at 5% (*p* > 0.05) * = Significant at 5% (*p* < 0.05).

*In bold, categories in which significance is appreciated (residual ≥ 2).*

In the results of the comparison between uses of music during and before confinement (Table 3) only one variable, singing, was not statistically significant. However, significant results with small effect sizes were observed for some musical practices, such as listening to music, playing an instrument, or dancing.

The perceptions of the importance of music in lockdown (Table 4) varied according to the respondents’ employment situation. We found high statistical significance in all variables, some with moderate (e.g., greater connection with others) and notable (e.g., helps you cope with the situation) effect sizes. These results therefore provide solid evidence of the effect of the employment factor. Our analysis of the mean values revealed that these differences follow the same pattern as the previous items: the retirees have the lowest perception of the value of music as compared to the other groups.

**TABLE 4:** Comparative inferential analysis: Perceptions of the impact of music (I).

*VARIABLES/Categories*	*Percentage*					*One-way ANOVA*		Effect size
		
	Unemployed	Furloughed	Retiree	Teleworking	Face-to-face working	Value	*P* value	
***HELPS YOU RELAX***	4.16	4.36	**3.59**	4.37	4.25	22.70**	<0.001	0.057
(*sample sizes*)	*n* = 171	*n* = 112	*n* = 130	*n* = 920	*n* = 179			
*% of categ. A LOT*	48.5%	53.6%	26.2%	57.4%	51.4%			
***HELPS YOU ESCAPE***	4.22	4.38	**3.56**	4.40	4.33	24.37**	<0.001	0.061
*(sample sizes)*	*n* = 169	*n* = 114	*n* = 122	*n* = 918	*n* = 177			
*% of categ. A LOT*	55.6%	56.1%	23.8%	59.0%	54.2%			
***IMPROVES MOOD***	4.37	4.49	**3.61**	4.54	4.46	37.64**	<0.001	0.091
*(sample sizes)*	*n* = 172	*n* = 113	*n* = 129	*n* = 918	*n* = 178			
*% of categ. A LOT*	61.6%	61.9%	30.2%	66.2%	62.9%			
***KEEPS YOU COMPANY***	4.25	4.32	**3.59**	4.45	4.41	28.62**	<0.001	0.071
*(sample sizes)*	*n* = 170	*n* = 114	*n* = 131	*n* = 915	*n* = 176			
*% of categ. A LOT*	56.5%	49.1%	30.5%	61.9%	60.8%			
***HELPS YOU COPE BETTER WITH CONFINEMENT***	4.09	4.26	**3.44**	4.31	4.18	23.60**	<0.001	0.059
*(sample sizes)*	*n* = 169	*n* = 116	*n* = 132	*n* = 917	*n* = 176			
*% of categ. A LOT*	46.2%	49.1%	22.7%	55.5%	47.7%			
***BOOSTS CONFIDENCE/POSITIVITY***	4.15	4.22	**3.41**	4.29	4.22	22.55**	<0.001	0.057
*(sample sizes)*	*n* = 169	*n* = 113	*n* = 122	*n* = 914	*n* = 176			
*% of categ. A LOT*	50.3%	47.8%	18.9%	54.3%	50.0%			
***IMPROVES CONNECTION WITH OTHERS***	4.03	4.10	**3.25**	4.20	4.16	20.12**	<0.001	0.052
*(sample sizes)*	*n* = 165	*n* = 110	*n* = 106	*n* = 909	*n* = 172			
*% of categ. A LOT*	45.5%	43.6%	17.0%	51.3%	47.7%			

** = Highly significant at 1% (*p* < 0.01).

*In bold, average values that are significantly lower.*

Finally, the intersection with information from the second group of questions on the perceptions of the value of music revealed similar results (Table 5). The retirees scored lower average values. However, they were not statistically significant in all variables: neither the value of musicians’ work nor the role of music in education had significant values. The following variables were significant: (1) the value of music, (2) music enriches free time, and (3) the influence of music on personal wellbeing.

**TABLE 5:** Comparative inferential analysis: Perceptions of the impact of music (II).

*VARIABLES/Categories*	*Percentage*					*One-way ANOVA*		Effect size
		
	Unemployed	Furloughed	Retiree	Teleworking	Face-to-face working	Value	*P* value	
***THE VALUE OF MUSIC***	3.49	3.62	**3.13**	3.54	3.43	3.03 *	0.017	0.008
*(sample sizes)*	*n* = 169	*n* = 113	*n* = 124	*n* = 911	*n* = 175			
*% of categ. A LOT*	29.6%	38.1%	19.4%	33.4%	26.9%			
***THE VALUE OF THE WORK OF MUSICIANS***	3.66	3.71	3.34	3.63	3.53	1.85 ^NS^	0.120	0.005
*(sample sizes)*	*n* = 164	*n* = 113	*n* = 129	*n* = 911	*n* = 175			
*% of categ. A LOT*	33.5%	43.4%	24.8%	35.8%	28.6%			
***THE ROLE OF MUSIC IN EDUCATION***	3.69	3.72	3.37	3.67	3.54	1.90 ^NS^	0.109	0.005
*(sample sizes)*	*n* = 164	*n* = 114	*n* = 127	*n* = 914	*n* = 176			
*% of categ. A LOT*	41.5%	42.1%	25.2%	38.4%	29.0%			
***MUSIC ENRICHES FREE TIME***	3.87	3.94	**3.47**	3.78	3.73	2.84 *	0.023	0.008
*(sample sizes)*	*n* = 167	*n* = 115	*n* = 129	*n* = 916	*n* = 175			
*% of categ. A LOT*	41.9%	47.0%	25.6%	40.7%	32.6%			
***INFLUENCE OF MUSIC ON PERSONAL WELLBEING***	3.86	3.93	**3.44**	3.81	3.72	3.30 *	0.011	0.009
*(sample sizes)*	*n* = 168	*n* = 114	*n* = 133	*n* = 918	*n* = 177			
*% of categ. A LOT*	41.7%	46.5%	28.6%	41.4%	35.0%			

N. S. = Not significant at 5% (*p* > 0.05) * = Significant at 5% (*p* < 0.05).

*In bold, average values that are significantly lower.*

## Discussion

COVID-19 has greatly impacted musical activities and, in many cases, has driven a decline of certain forms of musical consumption, first because the majority of concerts and cultural events have been cancelled during the pandemic (Agarwal and Sunitha, 2020), second due to the decrease of music in public spaces (Botstein, 2019), and third as a result of the decline in digital consumption of music (Sim et al., 2020), in many cases linked to people’s mobility and transit time or the time spent on retail and recreation, in parks, transit stations, and in workplaces.

Following methodological approaches to studying the use of music in people’s everyday lives (Craft et al., 1993; DeNora, 2000; North et al., 2000, 2004; Schäfer et al., 2013) the results of this study draw on testimonies of the perceived uses people make of music, and also the degree of impact they believe these activities have on their everyday lives. The data analyzed in this study reports subjective perceptions of the time people claim to devote to musical activities, such as listening, performing a musical instrument, singing or dancing.

According to the study’s findings, the Spanish population has turned to music during the present lockdown. The values in our results on uses of music, in the section analyzing musical identities in lockdown, reveal that a large percentage of the sample reported spending more time to music listening and making and show a keen interest in participating in cultural activities, specifically those of a musical nature.

Compared with previous research on the uses of music, Rentfrow and Gosling (2003) found that people reported listening to music most regularly while traveling, followed by alone at home. Music consumption in these cases correlates strongly with background use of music (Chamorro-Premuzic et al., 2012) and in most cases it occurs outside the home. These results are consistent with the aforementioned study of Sim et al. (2020), who analyze the decrease in digital consumption of music during the pandemic. However, when the consumption of music occurs in public environments or as background music (Chamorro-Premuzic and Furnham, 2007) people are less engaged with the music and have less intentional control over what they are listening to. In these cases, therefore, the usefulness of music for fulfilling logical mood management strategies may be not so obvious (North et al., 2004). This in turn affects people’s perceptions of their conscious use of music. Indeed, a previous analysis of the uses of music in everyday life (North et al., 2004) indicates that intentional exposure to music occurs mainly in the home. Our results coincide with this conclusion and indicate that as the amount of time spent at home increases, the perception of the intensity of the use of music also rises. At the same time, it is interesting to note that during confinement, while still remaining faithful to their musical preferences, a large number of people investigated new groups and styles of music. Discovering new music generates pleasure and is a stimulating choice. Variety, surprise and the discovery of the unknown are therefore essential factors in the enjoyment and appreciation of music (Hagendoorn, 2003). Devoting time and effort to explore new art has positive benefits (Tepper and Hargittai, 2009), which becomes more evident in difficult situations such as confinement.

During socially challenging circumstances, there is a practically unanimous consensus that music can be beneficial. Musical initiatives during the period of confinement, by both professional and amateur musicians, have fundamentally aspired to share music and raise the spirits of neighbors and society in general. These initiatives reflect the widespread perception among citizens that making music for other people contributes to their personal wellbeing (Daykin et al., 2018). In this way, the new musical initiatives to emerge in this period are essentially social in character, and go beyond aesthetic enjoyment. One heartening result to come out of this study, according to the analysis of the uses and knowledge of COVID-19 musical initiatives, is that practically all the survey respondents (93.4%) were aware of the musical initiatives for social purposes undertaken during confinement, and more than half (55.3%) followed or actively participated in them. Also, more than half of the surveyed population (53.2%) live in neighborhoods where residents have shared music on their balconies, and a quarter of them were actively involved in some of these proposals. These findings show that sharing music and musical practices is seen as an act of altruism and solidarity (Laurence and Urbain, 1999; Fukui and Toyoshima, 2014).

The data presented in this article show that Spanish society has a strikingly high perception of the value of music. The third section of the survey, perceptions on the impact of music, reveal that the perceived positive benefits of participating in artistic and musical activities for health, care and wellbeing, previously reported by health professionals (Wilson et al., 2016), is widely shared by society in general. Indeed, less than 1% of the sample perceived no benefit from such participation. The perception of the value of engaging in participatory musical activities has been studied in a range of settings (Kokotsaki and Hallam, 2007), and the literature reports benefits that coincide with this research: social improvements, such as a sense of belonging (Schäfer et al., 2013; Schäfer and Eerola, 2020) or altruism (Fukui and Toyoshima, 2014), and personal improvements, such as trust (Anshel and Kipper, 1988), positivity and mood regulation (Schäfer et al., 2013). Our results seem to suggest that the positive attributes of music were perceived as very high during lockdown. In addition, they show a rise in the value society now places on the social role of music, musicians and music education.

Although the conclusions drawn from the data are, in general, positive, this study also finds that people’s current employment situation has a significant direct influence on the uses and perception of the value of music. The groups that most reported listening to more music than before were the furloughed or teleworkers, while the unemployed and face-to-face workers claimed to listen to the same amount of music as before. This finding concurs with North et al. (2004) idea that most music is consumed at home, so those whose situation forced them to spend more time at home were consuming more music, while people whose day-to-day working practices remained the same did not change their cultural consumption behaviors to the same extent.

Although the pandemic is having a considerable impact for everyone in Spain, COVID-19 does not affect all citizens in the same way, as some people are more vulnerable to its effects on health, work activity, or individual or household economy. This differentiation has had a direct impact on the uses of music.

As a consequence of the health crisis, a large number of people are not in active work; many have been temporarily furloughed, and their return to work is dependent on economic recovery after the crisis (National Statistics Institute of Spain, 2020). It is precisely this group who, during confinement, have spent more of their time on musical activities such as listening, dancing or playing an instrument. These are also the people who have devoted most time to discovering new musical styles and groups, and are benefiting psychologically from exploring and finding new music (Hagendoorn, 2003; Tepper and Hargittai, 2009).

The oldest group in the sample, the retirees, differed in both their uses of music and their perceptions of its value. The elderly have been severely impacted by the pandemic to the point that those over the age of 65 account for 90.7% of deaths from COVID-19 in Spain (Spanish Ministry of Health, 2020). This situation inevitably causes a great deal of stress and feeling of vulnerability among the population. As a result, older people are generally dealing with stricter levels of isolation and, therefore, greater loneliness. As a protective measure against contagion, they are forced to stay at home and have little or no interaction with other people. Several studies have analyzed the age factor in aspects related to music, such as musical preferences (LeBlanc et al., 1996; Bonneville-Roussy et al., 2013), individual differences in music consumption (Chamorro-Premuzic et al., 2012), the perception of musical mode and rhythm (Halpern et al., 1998) or emotional judgments of music (Vieillard et al., 2012). Although previous studies have explored perceptions of the value of music among vulnerable people (O’Callaghan et al., 2014), its influence on the most vulnerable age group to the disease had not, to date, been examined.

In this case, our data reveal that older people in vulnerable situations spend time listening to music. Compared with studies that analyze how frequently older people listen to music in other countries (Laukka, 2007), Spanish people reported more frequent everyday music listening during the pandemic, although their use is less frequent than other age groups. They are also the group that has least increased the time they devoted to musical practices during lockdown. In a previous study, Chamorro-Premuzic et al. (2012) also identified age as a factor affecting significant effects of all music uses on music consumption. Likewise, the time they devote to musical practices is spent listening to and performing music that is already part of their cultural background and musical preferences, rather than discovering and getting to know other forms or musical styles. Finally, older people had a more positive view of the impact of music in personal wellbeing compared to previous studies (Laukka, 2007), particularly in aspects such as the power of music to improve mood, reduce feelings of loneliness, or encourage feelings of similarity and connection with others. However, although they scored positively, this group presents the least favorable data in the perception of the potential of music to contribute to psychological and social wellbeing. Chamorro-Premuzic et al. (2012) analyzed age as a factor influencing the emotional uses of music. Their findings supported those of a previous study by Chamorro-Premuzic et al. (2009), which found negative correlations between age and the emotional use of music in a Spanish study. In relation to the use of music for emotional regulation, our study presents new analysis of the perception of the value of music for psychological wellbeing, providing evidence of a relationship between these perceptions and conditions such as age or feelings of vulnerability. Our survey data showed the greatest divergence among the group of retired people and, consistent with the aforementioned studies (Chamorro-Premuzic et al., 2009, 2012), older Spanish people scored lower in all the dimensions analyzing the perception of the value of music for wellbeing and positive emotions. In addition, our study goes a step further by showing that this group reported the lowest scores on the overall impact of the value of music to positively affect people’s lives during the pandemic.

In conclusion, the study reveals that music has played an important role in people’s lives during the lockdown and that aspects such as having more time at home, willingness to help others, and the feeling of facing a collective social problem, increase the use of musical practices and raise perceptions about the value of music in psychological and social wellbeing. However, age and, more specifically, the feeling of isolation or sense of vulnerability, can lead to more conservative use of musical practices and moderate perceptions of the positive value of music.

This research paints a broad picture of the uses and perceptions of music during lockdown, and invites reflections on how a major change in people’s everyday lives can influence their relationship with musical phenomena. The findings suggest several directions for future research.

First, although the number of participants reliably represents the target population, the sample is not internally representative of the Spanish population in strata such as gender or level of studies and could, therefore, introduce bias in analyzing patterns of uses and influence of music according to factors such as gender, level of studies or sociodemographic groups. Furthermore, the absence of a control group represents a methodological limitation in understanding actual use of music before and during the pandemic. As there are no previous studies analyzing the same aspects of music making and the perception of the value of music in psychological wellbeing among the Spanish population, it is difficult to observe causal directions of the pandemic in any dimensions of the findings. We believe that conducting similar research after the pandemic will offer new interesting information to map the actual effect the lockdown had on the use of music in everyday life. Lastly, we did not obtain data about the influence music has in children’s lives during lockdown. Now we are aware of the psychological effects the pandemic is having on children’s lives (Jefsen et al., 2020; Phelps and Sperry, 2020; Valenzuela et al., 2020), it is important to conduct further research in this area.

Second, we agree with North et al. (2004) that the respondents’ interpretations of their uses and perceptions of music may lead to inaccurate responses. We are aware that, in certain cases, the idea people have about the intention of and attention to their participation in music is not always unequivocally linked to the amount of time they actually devote to music listening or making. We consider that the importance of analyzing people’s perception of their use of music relies on understanding the role of music in everyday life in terms of how it shapes and reflects an individual’s identity; in considering music in everyday life as a way of organizing one’s internal and social world. And this, as the sociologist Frith (1996) argues, comes from the “imagined self.” However, we believe that future research could usefully enrich this analysis by taking a complementary experimental approach.

## Data Availability Statement

The raw data supporting the conclusions of this article will be made available by the authors, without undue reservation.

## Ethics Statement

Ethical review and approval was not required for the study on human participants in accordance with the local legislation and institutional requirements. The patients/participants provided their written informed consent to participate in this study.

## Author Contributions

All the authors equally collaborated in designing the research, collecting and analyzing the data, and wrote collaboratively in writing the manuscript.

## Conflict of Interest

The authors declare that the research was conducted in the absence of any commercial or financial relationships that could be construed as a potential conflict of interest.
