# Supplementary material for: Uses and Perceptions of Music in Times of COVID-19: A Spanish Population Survey
Source: Front Psychol. 2021 Jan 12;11:606180. doi: 10.3389/fpsyg.2020.606180 (PMC7835488; doi:10.3389/fpsyg.2020.606180)
Supplement: Supplementary file 1 [file Data_Sheet_1.PDF]

MUSIVID19

Estimado/a,

Somos un grupo de investigadores/as pertenecientes a los grupos de investigación "Educación, Arte y Sociedad" de la Universidad Jaume I de Castellón y la "KideOn" de la Universidad del País Vasco. En el marco del proyecto de investigación con referencia EDU2017-86311-P, financiado por el Ministerio de Ciencia e Innovación, estamos llevando a cabo un estudio con objeto de analizar el papel de la música en esta situación de confinamiento relacionado con el COVID-19.
En cumplimiento de lo dispuesto en el Reglamento (UE) 2016/679, General de Protección de Datos de Carácter Personal, le informamos que los datos de carácter personal obtenidos mediante la cumplimentación del presente formulario van a ser tratados con su consentimiento, de manera anónima y para la exclusiva finalidad de promoción y divulgación científica. Le agradecemos que conteste a la siguiente encuesta con sinceridad y que nos ayude con su difusión.

Completar la encuesta le llevará 5 minutos aproximadamente.

Muchas gracias de antemano.

Dear Sir or Madam,
We are researchers from the research groups "Education, Art and Society" at the University Jaume I of Castellón and the "KideOn" at the University of the Basque Country. Within the framework of the research project reference EDU2017-86311-P, funded by the Ministry of Science and Innovation, we are carrying out a study to analyse the role of music in this situation of COVID-19-related lockdown.
In compliance with the provisions of the EU General Data Protection Regulation 2016/679, we inform you that the personal data obtained by completing this form will be treated with your consent, anonymously and for the exclusive purpose of promotion and scientific dissemination. We would be grateful if you would answer the following survey questions honestly and help us with its dissemination.

Completing the survey will take approximately 5 minutes.

Thank you very much in advance.

1. Consentimiento informado / *Informed consent*

Mark only one box.

- ◯ Soy mayor de edad y deseo participar en el estudio / *I am of legal age and wish to participate in the study*
- ◯ No soy mayor de edad o no deseo participar en el estudio */ I am not of legal age or do not wish to participate in the study*

2. Género / *Gender*

Mark only one box.

- [ ] Mujer / *Female*
- [ ] Hombre / *Male*
- [ ] Otros / *Other*: ____________________

3. Edad / *Age*

____

4. Provincia de residencia / *Province of residence*

Mark only one box.

- A Coruña (La Coruña)
- Álava (Araba)
- Albacete
- Alicante
- Almería
- Asturias
- Ávila
- Badajoz
- Barcelona
- Burgos
- Cáceres
- Cádiz
- Cantabria
- Castellón
- Ciudad Real
- Córdoba
- Cuenca
- Girona (Gerona)
- Granada
- Guadalajara
- Guipúzkoa
- Huelva
- Huesca
- Islas Baleares
- Jaén
- La Rioja
- Las Palmas
- León
- Lérida (Lleida)
- Lugo
- Madrid
- Málaga
- Murcia
- Navarra
- Orense (Ourense)
- Palencia
- Pontevedra
- Salamanca
- Segovia
- Sevilla
- Soria
- Tarragona
- Tenerife
- Teruel
- Toledo
- Valencia
- Valladolid
- Vizcaya (Bizkaia)
- Zamora
- Zaragoza
- Ciudades autónomas de Ceuta y Melilla

5. Nacionalidad / *Nationality* *

Mark only one box.

- Española / *Spanish*
- Otra / *Other*:………………..

6. ¿Vive acompañado? / *Do you live with other people?*

Mark only one box.

- Sí / *Yes*
- No / *No*

7. En caso afirmativo, ¿vive con algún menor? / *If so, do you live with anyone under the age of 18?*

Mark only one box.

- Sí / *Yes*
- No / *No*

8. Profesión / *Profession*

____

9. Actualmente está... / *Currently, you are…*

Mark only one box.

- Teletrabajando / *Teleworking*
- Trabajando de manera presencial / *Face-to-face work*
- En régimen de expediente de regulación temporal de empleo (ERTE) / *Furloughed*
- Desempleado/a / *Unemployed*
- Vacaciones / *On holiday*
- Jubilado/a / *Retired*
-

Ninguno de los anteriores / *None of the above*

10. Nivel de estudios / *Level of studies*

Mark only one box.

- Sin estudios reglados / *No formal studies*
- Educación primaria / *Primary Education*
- Educación secundaria / *Secondary Education*
- Estudios universitarios / *University Education*
- Otros / *Others*: ______________________

DURANTE EL PERIODO DE CONFINAMIENTO... / *DURING THE LOCKDOWN PERIOD….*

11. ¿Con qué frecuencia escucha música? / How often do you listen to music?

Mark only one box.

- Todos los días / *Every day*
- De 3 a 5 días por semana / *3-5 days per week*
- Entre 1 y 2 días por semana / *1-2 days per week*
- Menos de una vez a la semana */ Less than once per week*

12. Si ha seleccionado "Todos los días", ¿cuántas horas al día aproximadamente? / *If you selected "Every day", how many hours approximately?*

Mark only one box.

- Más de 5 horas / *More than 5 hours a day*
- Entre 3 y 5 horas / *3-5 hours a day*
- Entre 1 y 2 horas / *1-2 hours a day*
- Menos de 1 hora / *Less than 1 hour a day*

13. Durante este periodo, ¿ha escuchado una mayor diversidad de música? */ During this period, have you listened a greater diversity of music?*

Mark only one box.

- Sigo escuchando la misma música que antes / *I listen to the same music as I did before*
- He descubierto nuevos estilos musicales / *I have discovered new musical styles*

14. ¿Ha descubierto nuevos grupos musicales que son de su agrado durante este periodo? / Have you discovered new musical groups that you like during this period?

Mark only one box.

- Sí / *Yes*
- No / *No*

15. ¿Conoce iniciativas o proyectos que han realizado músicos para compartir o contribuir al bienestar durante este periodo de confinamiento? / *Do you know of any initiatives or projects that musicians have undertaken to share or contribute to wellbeing during this period of confinement?*

Mark only one box.

- Conozco y he lanzado o participado en alguna de ellas / *I know about and have instigated or participated in them*
- Conozco algunas iniciativas y las sigo con entusiasmo / *I know about them and follow them enthusiastically*
- He escuchado que algunos músicos han lanzado proyectos / *I have heard about them*
- No conocía este tipo de iniciativas / *I have no knowledge of them*

16. ¿Aprovecha las iniciativas solidarias y creativas que están lanzando a través de plataformas los músicos o asociaciones musicales? Señale cuáles. / *Do you participate in supportive and creative initiatives that musicians or musical associations are undertaking?*

Tick all that apply.

- [ ] No he participado en ninguna de ellas / *I haven’t participated in any*
- [ ] Conciertos en "streaming" / *Streamed concerts*
- [ ] Festivales en línea / *Online festivals*
- [ ] Acceso abierto a plataformas de contenido musical / *Open access to musical platforms*
- [ ] Campañas musicales / *Musical campaigns*
- [ ] Clases de música gratuitas en línea / *Free online music lessons*
- [ ] Acciones de creación musical colaborativa / *Collaborative music creation actions*
- [ ] Retos musicales / *Musical challenges*
- [ ] Otros / *Others*: ____________________

17. En su barrio, ¿hay vecinos/as haciendo música desde los balcones? / *In your neighbourhood, are people making music on their balconies?*

Mark only one box.

- Sí / *Yes*
- No / *No*

18. En caso afirmativo, ¿qué hace usted en esos momentos? / *If so, what do you do at those times?*

Tick all that apply.

- [ ] Soy yo quien hago música desde los balcones / *I make music on my balcony*
- [ ] Acompaño con palmas / *I accompany with clapping*
- [ ] Bailo / *I dance*
- [ ] Escucho la música / *I listen to the music*
- [ ] No participo / *I do not participate*
- [ ] No me gusta que hagan música en los balcones / *I do not like it when people make music on their balconies*
- [ ] Otros / *Others*: ____________________

19. Con respecto a antes de la situación de confinamiento, considera que... / Compared to the period before the lockdown do you think you…

Mark only one box per row.

	Menos que antes / *Less than before*	Igual que antes / The same as before	Más que antes / *More than before*
...escucha música / *listen to music*			
...canta / *sing*			
...baila / *dance*			
...toca un instrumento / *play an instrument*			
...escucha otros estilos musicales / *listen to other musical styles*			

20. ¿Cuáles son sus percepciones acerca del valor de la música en estos momentos? / *What is your perception of the value of music at this time?*

Tick only one box per row.

	Nada / *Not at all*	Un poco / *A little*	A veces / *Sometimes*	Bastante / *Often*	Mucho / *A lot*
¿Le ayuda a relajarse? / *Helps you relax?*					
¿Le ayuda a evadirse? / *Helps you escape?*					
¿Le anima? / *Improves your mood?*					
¿Le hace compañía? / *Keeps you company?*					
¿Le ayuda a llevar mejor la situación de confinamiento? / *Helps you cope better with lockdown?*					
¿Le genera más confianza o positivismo? / *Boosts your confidence or positivity?*					
¿Le hace sentirse más conectado con otras personas? / *Improves connection with others?*					

21. ¿Cree que la situación de confinamiento ha mejorado su percepción acerca de...? / *Do you think confinement has improved your perception of...?*

Tick only one box per row.

	1	2	3	4	5
El valor de la música / *The value of music*					
El valor del trabajo de los músicos / *The value of musicians' work*					
El papel de la música en la educación / *The role of music in education*					
Las posibilidades de la música para enriquecer el tiempo libre? / *how music can enrich your free time?*					
La influencia de la música en el bienestar personal / *the influence of music on personal wellbeing*					

¡MUCHAS GRACIAS! / *THANK YOU VERY MUCH!*
